# Causal Relationships Between Epilepsy, Anti‐Epileptic Drugs, and Serum Vitamin D and Vitamin D Binding Protein: A Bidirectional and Drug Target Mendelian Randomization Study

**DOI:** 10.1111/cns.70183

**Published:** 2024-12-20

**Authors:** Zizhang Cheng, Jinyi Zuo, Xintao Peng, Haoran Zhang, Wenlong Su, Guoming Luan, Yuguang Guan

**Affiliations:** ^1^ Department of Neurosurgery, SanBo Brain Hospital Capital Medical University Beijing China; ^2^ Beijing Key Laboratory of Epilepsy Beijing China; ^3^ Center of Epilepsy, Beijing Institute of Brain Disorders, Collaborative Innovation Center for Brain Disorders, Laboratory for Clinical Medicine Capital Medical University Beijing China

**Keywords:** anti‐epileptic drug, epilepsy, Mendelian randomization, vitamin D, vitamin D binding protein

## Abstract

**Aims:**

Previous studies suggest potential associations between epilepsy, anti‐epileptic drugs (AEDs), and levels of vitamin D and vitamin D‐binding protein (VDBP). This study aims to investigate the causal relationships among these variables using Mendelian Randomization (MR) methods.

**Methods:**

Using summary data from genome‐wide association studies on serum 25‐hydroxyvitamin D [25(OH)D] levels (*N* = 417,580), VDBP concentrations (*N* = 65,589), and various types of epilepsy (Ncases = 27,559), MR analyses were conducted to determine bidirectional causal relationships among these variables. Additionally, eQTL data from eQTLGen (*N* = 31,684) were employed to model the effects of AEDs and evaluate their causal impact on both biomarkers.

**Results:**

No causal relationships were found between serum 25(OH)D or VDBP levels and epilepsy. Although genetically predicted focal epilepsy risk was potentially associated with increased serum 25(OH)D levels (OR 1.031, 95% CI: 1.006–1.058, *p* = 0.017), and a higher genetic risk of juvenile myoclonic epilepsy was linked to lower VDBP levels (OR 0.977, 95% CI: 0.961–0.993, *p* = 0.004), both associations lost significance after multiple correction. Furthermore, significant associations were observed between serum 25(OH)D levels and AED target genes SCN4A, GABBR1, CA13, ALDH5A1, and CA8. No significant associations were found between AED target genes and VDBP levels after correction.

**Conclusion:**

No causal relationships were found between genetically determined serum 25(OH)D levels, VDBP, and epilepsy or its subtypes. Furthermore, the use of AEDs, such as Carbamazepine, Oxcarbazepine, Progabide, and Valproic Acid, reduces serum 25(OH)D levels, while not affect VDBP levels.

## Graphical abstract

The study utilized single nucleotide polymorphisms from genome‐wide association studies as instrumental variables and employed Mendelian Randomization to uncover causal relationships between epilepsy and both vitamin D and vitamin D‐binding protein. Additionally, the findings support that anti‐epileptic drugs reduce serum vitamin D levels without affecting vitamin D‐binding protein.

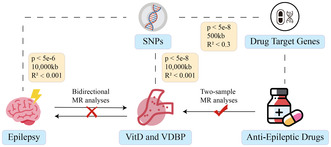

## 1 Introduction

Epilepsy is a chronic neurological disorder characterized by recurrent seizures, affecting more than 50 million individuals worldwide [1]. Despite significant advances in our understanding, approximately 50% of epilepsy cases remain with an unclear etiology [2, 3]. Observational studies show that individuals with epilepsy often have lower vitamin D (VitD) levels compared to the general population [4, 5, 6, 7]. However, these studies are frequently susceptible to confounding factors and reverse causation. Recent double‐blind randomized controlled trials investigating the impact of VitD on epilepsy outcomes have not demonstrated efficacy during the blinded phase, although some improvement in seizure control was observed in the subsequent open‐label phase [8]. Consequently, the question of whether VitD deficiency is a causal factor for epilepsy or a consequence of the condition remains debated.

VitD binding protein (VDBP) is the principal carrier of VitD in the bloodstream, transporting it to various tissues and organs. Research shows that VDBP levels are positively correlated with VitD levels [9, 10], and deficiencies in VDBP can lead to vitamin D deficiency [11]. Additionally, VDBP may also play a role in epilepsy [12]. Studies have found elevated VDBP levels in cerebrospinal fluid of patients with temporal lobe epilepsy [13], and genetic polymorphisms in VDBP may affect both seizure occurrence and epilepsy progression [14]. Given the potential links between VitD and epilepsy, it is crucial to further investigate the role of VDBP in the development of epilepsy.

Anti‐epileptic drugs (AEDs) are fundamental in treating epilepsy, but their long‐term effects on VitD metabolism and VDBP levels have not been extensively studied. Some AEDs may be associated with an increased risk of VitD deficiency [15, 16]. Additionally, evidence suggests that a greater number of AEDs prescribed may be linked to lower VitD levels in patients [16]. However, other studies indicate that neither monotherapy nor combination therapy with AEDs consistently lowers baseline serum VitD levels in epilepsy patients, suggesting that low VitD levels may be more closely related to the disease itself rather than the treatment [17].

Mendelian randomization (MR) uses genetic variants as proxies to infer causal relationships between exposures and outcomes [18], thereby reducing confounding factors and reverse causation commonly encountered in observational studies [19, 20]. This MR study aims to explore the bidirectional causal relationships between epilepsy, VitD, and VDBP, as well as to investigate whether AEDs act as independent factors affecting VitD and VDBP levels. By addressing these key issues, we seek to elucidate the complex interactions between epilepsy, VitD, and VDBP, and provide more reliable evidence to inform clinical practice.

## 2 Materials and Methods

### 2.1 Study Design

**FIGURE 1:**
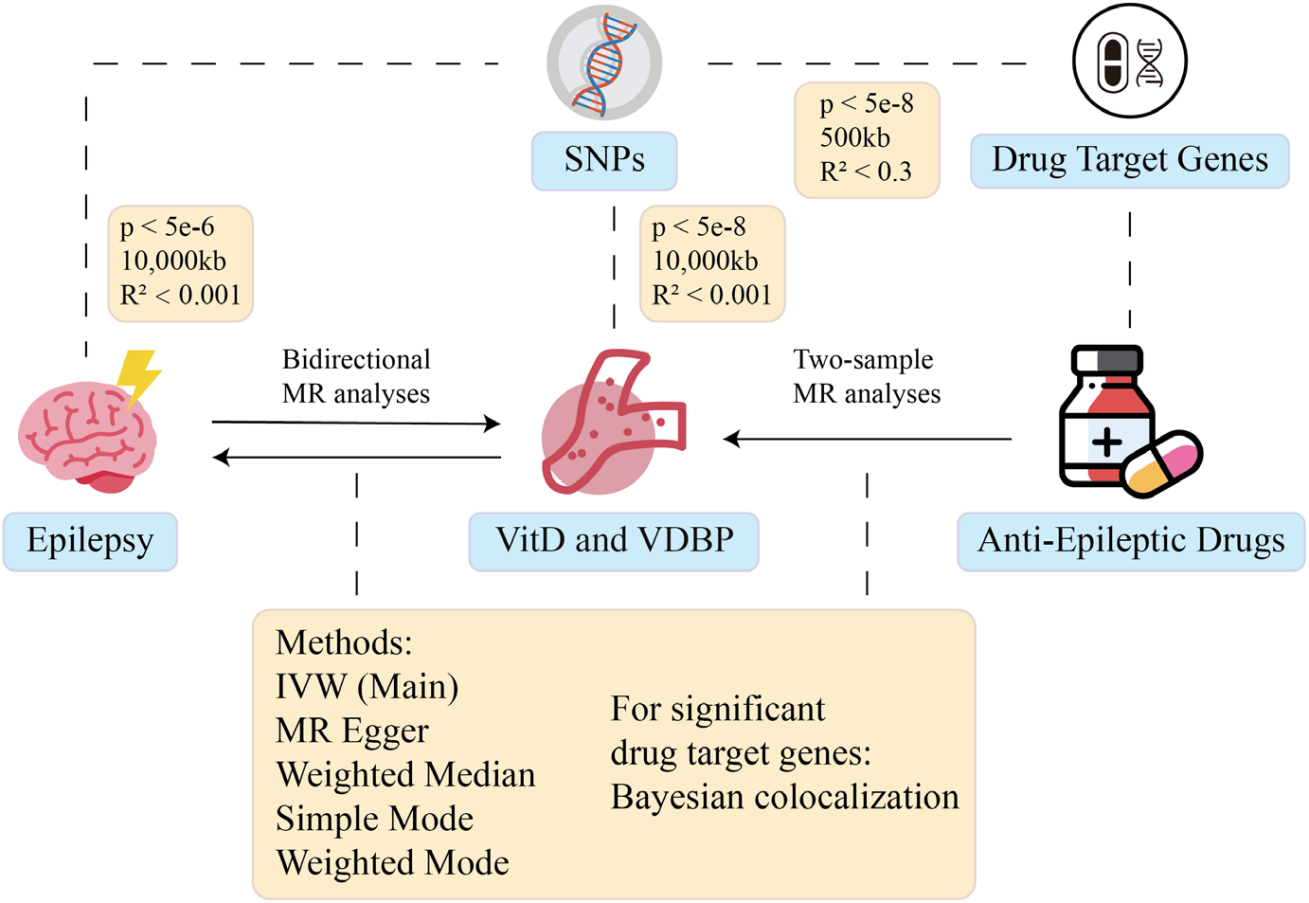
The flowchart of the study. This study employs bidirectional MR to evaluate the reciprocal causal effects between epilepsy and serum levels of VitD and VDBP. SNPs associated with the target genes of AEDs were extracted and used in two‐sample MR analyses to explore their causal relationships with serum vitamin D and VDBP levels. IVW, inverse variance weighted; MR, Mendelian randomization; SNP, Single nucleotide polymorphism; VDBP, vitamin D binding protein; VitD, vitamin D.

This MR study consists of two analytical components (Figure 1). The first component includes two separate two‐sample bidirectional MR analyses to assess the causal effects of epilepsy on serum vitamin D and VDBP levels, and vice versa. The second component focuses on the target genes of AEDs. Using eQTL data, we identified candidate genetic variants for each AED target gene and performed two‐sample MR to explore their causal relationships with VitD and VDBP levels. Additionally, colocalization analyses were performed on significant target genes to examine shared genetic influences between AED targets and these biomarkers.

This study utilizes data from public databases. All data used in this research underwent the necessary review and approval processes, and informed consent was obtained from all participants. Separate approval from an institutional review board was not required for this study.

### 2.2 Genetic Instruments Selection and Data Sources

25‐hydroxyvitamin D [25(OH)D], the primary biomarker for assessing VitD levels, is commonly used to evaluate VitD status. For our study, we obtained data related to serum 25(OH)D from a large‐scale genome‐wide association study (GWAS) conducted by Revez et al., which included 417,580 participants of European descent [21]. Data on VDBP levels were derived from the study by Albiñana et al., which measured VDBP concentrations in dried blood spots from 65,589 newborns of European descent [22] (Table S1). Single nucleotide polymorphisms (SNPs) were selected based on genome‐wide significance (*p* < 5e‐8) and linkage disequilibrium (LD) thresholds of 10,000 kb and *R* ^2^ < 0.001, using data from the 1000 Genomes Project European panel [23].

The summary statistics for the GWAS of epilepsy cases with European ancestry were sourced from the International League Against Epilepsy (ILAE) consortium [24]. This dataset includes 27,559 epilepsy cases and 42,436 controls, categorized into genetic generalized epilepsy (GGE) and focal epilepsy subtypes. Among the focal epilepsy cases, there are individuals with focal epilepsy without a known lesion, those with hippocampal sclerosis (HS), and cases with non‐HS lesions. Within the GGE subtype, the cases are further subdivided into childhood absence epilepsy (CAE), juvenile absence epilepsy (JAE), juvenile myoclonic epilepsy (JME), and generalized tonic–clonic seizures (GTCS). Due to the unavailability of SNPs associated with many epilepsy phenotypes at the genome‐wide significance threshold of *p* < 5e‐8, we adjusted the p‐value threshold for SNP selection to *p* < 5e‐6. To account for linkage disequilibrium, we defined the threshold as *R* ^2^ < 0.001 and set the window size to 10,000 kb.

Based on the latest epilepsy treatment guidelines [25], a list of commonly used AEDs was compiled. The pharmacological targets of these drug classes were identified using the DrugBank [26] (https://www.drugbank.ca/), which provides information on the effects of these drugs on their targets, such as agonists or inhibitors. To model the effects of AEDs, we utilized expression quantitative trait loci (eQTL) data to represent the action of these drugs through the expression of their target genes. This data was obtained from eQTLGen [27], a consortium comprising 37 datasets with a total of 31,684 individuals, reporting on the expression of 16,987 genes in whole blood. For the genetic instrumental variables associated with these targets, SNP selection was based on a p‐value threshold of 5e‐8, with clumping parameters set to 500 kb and *R* ^2^ < 0.3.

### 2.3 Statistical Analysis

In this study, we performed bidirectional MR analyses to examine the reciprocal causal relationships between epilepsy phenotypes and serum 25(OH)D and VDBP levels. Two‐sample MR analyses were used to explore the associations between AEDs and serum 25(OH)D and VDBP levels. MR is based on three core assumptions: (1) the genetic variants must be significantly associated with the exposure; (2) the association with the outcome must be independent of confounding factors; and (3) the effect on the outcome must be mediated exclusively through the exposure [18, 28].

The primary method employed was the random‐effects inverse variance weighted (IVW) approach, which accommodates potential heterogeneity in causal estimates [29]. To address possible heterogeneity and horizontal pleiotropy in the causal estimates, we supplemented the IVW analysis with MR Egger, weighted median, simple mode, and weighted mode methods, providing additional evidence for the causal relationships investigated. When only a single SNP was available, the Wald ratio method was used to assess the causal relationship between the variables [18, 30, 31]. We subsequently employed Bayesian colocalization methods to investigate whether the expression of positive drug target genes and serum 25(OH)D share common genetic variants within specified genomic regions [32]. A posterior probability greater than 80% is generally considered to provide strong support for the presence of shared causal variants. All the results are presented as odds ratios (OR) with 95% confidence intervals (CI).

In addition, we employed Linkage Disequilibrium Score Regression (LDSC) to evaluate genetic correlations between genes, a method that facilitates the understanding of shared genetic architectures in genome‐wide associations [33, 34]. This approach was used to explore the genetic correlation between the eQTLs of positive drug target genes. Furthermore, colocalization analysis was conducted to assess potential genetic colocalization between these eQTLs, thereby elucidating whether the effects of the drug targets on vitamin D are independent.

To assess the robustness of our results, we performed a series of sensitivity analyses. For each SNP, *R* ^2^ and F‐statistics were calculated to evaluate the explained variance and potential weak instrument bias. SNPs with an F‐statistic < 10 were considered weak instrumental variables and were excluded from the dataset [35]. Cochran's Q statistic was utilized within the IVW model to evaluate heterogeneity among the variance‐specific estimates. MR‐Egger regression intercept and Mendelian Randomization Pleiotropy RESidual Sum and Outlier (MR‐PRESSO) tests were employed to assess horizontal pleiotropy [18]. Additionally, leave‐one‐out analyses were performed to determine whether any single SNP was driving or biasing the causal estimates.

Bonferroni correction was applied to determine the threshold for statistical significance in all MR analyses. *p*‐values between 0.05 and the adjusted threshold were considered suggestive of potential associations. In the first component of the analysis, the threshold for each set of analyses was set at *p* < 0.05/20 (10 epilepsy and its subtypes × 2 directions). In the second component, the adjusted threshold was determined as *p* < 0.05/ the number of target genes analyzed involved in the analyses for each group. Two‐sample MR analyses were conducted using the TwoSampleMR package (version 0.6.4) and the MRPRESSO package (version 1.0) in R (version 4.4.1). Colocalization analyses were performed using the coloc R package (version 5.2.3).

## 3 Results

### 3.1 Epilepsy and Serum 25(OH)D, VDBP

**FIGURE 2:**
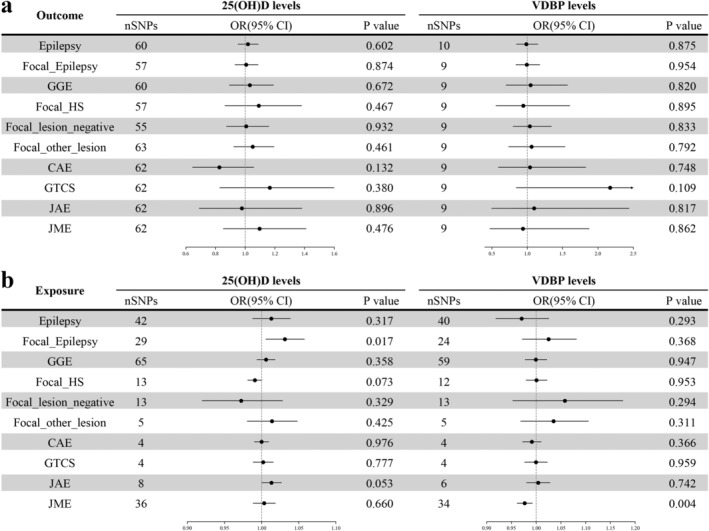
Association between genetically determined epilepsy and its subtypes risk with serum 25(OH)D and VDBP levels. (a) MR results showing the causal effects of serum 25OHD and DBP levels on the risk of epilepsy. (b) MR results illustrating the causal relationships of genetically predicted epilepsy risk on serum 25(OH)D and DBP levels25(OH)D 25‐hydroxyvitamin D; CAE, Childhood Absence Epilepsy; CI, confidence interval; Focal_HS, FocalEpilepsy, documented hippocampal sclerosis; Focal_Lesion_Negative, Focal Epilepsy, documented lesion negative; Focal_Other_Lesion, Focal Epilepsy, documented lesion other than HS; GGE, genetic generalized epilepsy; GTCS, generalized tonic–clonic seizures; JAE, juvenile absence epilepsy; JME, juvenile myoclonic epilepsy; MR, Mendelian randomization; OR, odds ratio; SNP, Single nucleotide polymorphism; VDBP, vitamin D binding protein.

In this MR study, we did not find evidence of a causal relationship between genetically determined serum 25(OH)D levels and epilepsy or its subtypes (Table S2). However, in the reverse MR analysis, there was a potential association between the genetically predicted risk of focal epilepsy and serum 25(OH)D levels (OR 1.031, 95% CI: 1.006–1.058, *p* = 0.017) (Figure 2a). This suggests that focal epilepsy may potentially lead to an increase in serum 25(OH)D levels. Nonetheless, this association lost significance after applying the Bonferroni correction.

The primary IVW analysis showed no evidence of a causal relationship between genetically determined VDBP concentrations and the risk of epilepsy or its subtypes (Figure 2b). In the reverse analysis, an initial finding suggested that a higher genetic predisposition to juvenile myoclonic epilepsy might be linked to lower VDBP levels (OR 0.977, 95% CI: 0.961–0.993, *p* = 0.004). However, this association did not withstand adjustment for multiple comparisons, rendering it statistically nonsignificant.

The F‐statistics of all genome‐wide significant SNPs used in the analysis were greater than 10 (Table S3). In the sensitivity analyses, some causal effects exhibited heterogeneity according to Cochran's Q test (Table S4). To address this issue, we employed IVW with a random effects model, which partially mitigated the observed heterogeneity. MR‐PRESSO analysis did not reveal evidence of directional pleiotropy. Furthermore, the MR‐Egger intercept and leave‐one‐out sensitivity analysis confirmed the robustness of all SNPs, demonstrating the stability of the results and indicating that no single SNP had a disproportionate impact on the findings.

### 3.2 AEDs And Serum 25(OH)D, VDBP

A total of 35 AEDs were included in the study, leading to the identification of 126 target genes associated with these drugs (Table S5). Among these genes, 65 were found to have strong associations with eQTL SNPs in the eQTLGen dataset (F‐statistic > 10, p‐eQTL < 5e‐8, Table S6). These genetic proxies were then used to investigate the effects of AEDs on serum 25(OH)D and VDBP levels (Table S7).

**FIGURE 3:**
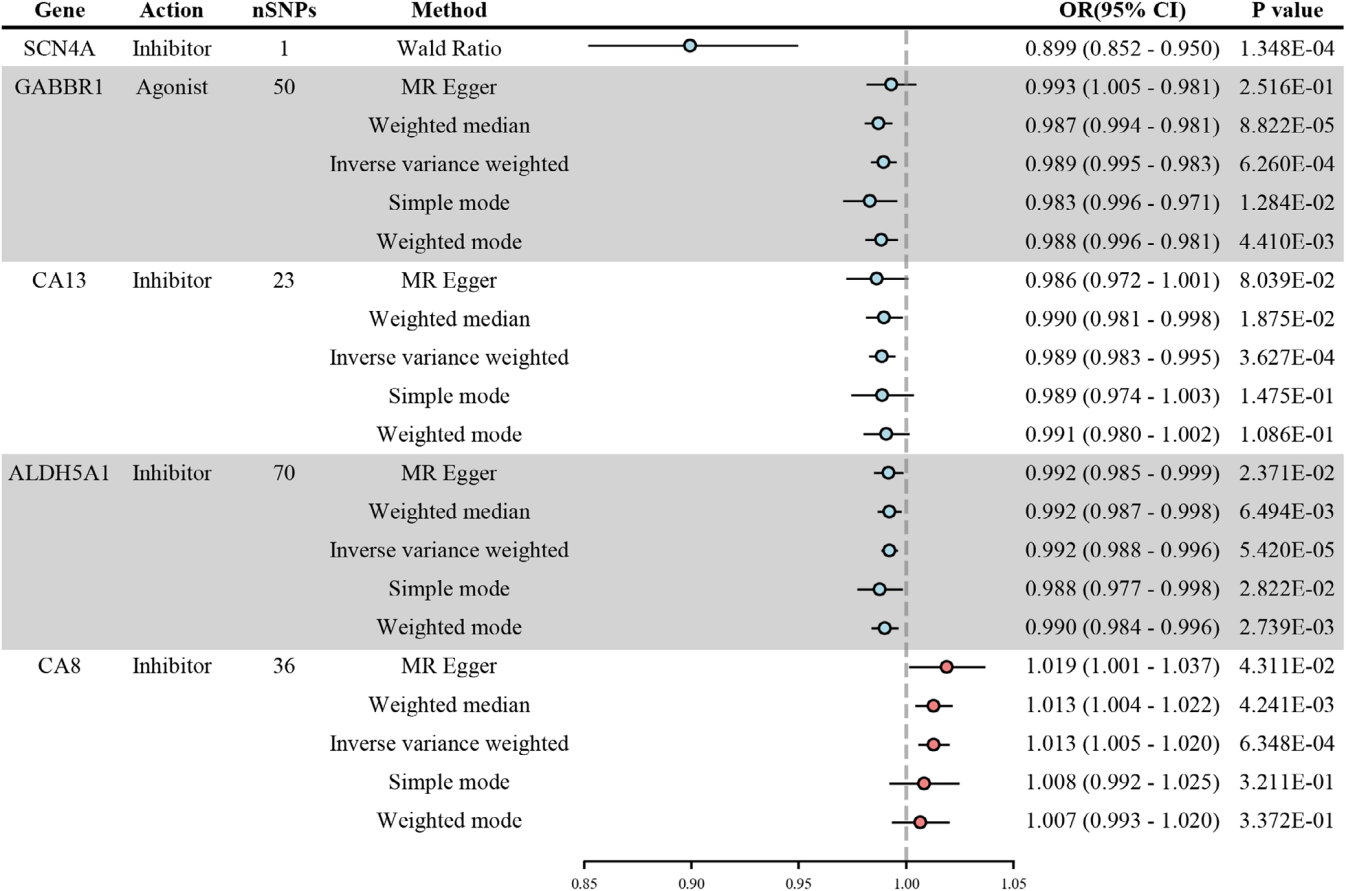
Significant associations between anti‐epileptic drug targets and serum 25‐hydroxyvitamin D levels. Causal estimates are shown as colored dots, with blue indicating a protective effect and red a promoting effect. CI, confidence interval; OR, odds ratio; MR, Mendelian randomization; SNP, single nucleotide polymorphism.

In the analysis of the effects of AEDs on VitD, 15 distinct genes were identified as being associated with serum 25(OH)D levels. After applying the Bonferroni correction, five of these target genes maintained significant relationships with serum 25(OH)D levels (Figure 3). Notably, 80% of the drug target genes (4 of 5) were found to be connected to lower serum 25(OH)D levels through their respective agonists or inhibitors. Specifically, the inhibitors of SCN4A were associated with a decrease in serum 25(OH)D levels (OR 0.899, 95% CI: 0.852–0.950, *p* = 1.348e‐4). Similarly, the agonist of GABBR1 showed a similar effect, correlating with a reduction in serum 25(OH)D levels (OR 0.989, 95% CI: 0.995–0.983, *p* = 6.260e‐4). The inhibitors of CA13 were linked to decreased serum 25(OH)D levels (OR 0.989, 95% CI: 0.983–0.995, *p* = 3.627e‐24), and the inhibitors of ALDH5A1 also showed a similar effect (OR 0.992, 95% CI: 0.988–0.996, *p* = 0.004). Conversely, the inhibitors of CA8 were found to be associated with an increase in serum 25(OH)D levels (OR 1.013, 95% CI: 1.005–1.020, *p* = 6.348e‐4).

In terms of specific medications, the use of Topiramate, Lamotrigine, Carbamazepine, Oxcarbazepine, and Brivaracetam (all SCN4A antagonists) as well as Progabide (a GABBR1 agonist) has been associated with a decrease in serum 25(OH)D levels. Additionally, Valproic Acid, which acts as both an SCN4A and ALDH5A1 antagonist, also showed a connection to lower serum 25(OH)D levels. In contrast, the impact of Zonisamide, which functions as an SCN4A antagonist, CA13 antagonist, and CA8 antagonist, remains inconclusive due to its multiple target interactions, making it difficult to determine its effect on serum 25(OH)D levels based solely on our analysis.

In the analysis of VDBP levels, three target genes related to AEDs were identified, each showing preliminary *p*‐values below 0.05. However, after applying the Bonferroni correction to account for Type I error risk, none of these genes demonstrated statistically significant associations with VDBP levels. Specifically, after adjustment, the *p*‐values for all these genes exceeded the threshold for statistical significance, suggesting that the impact of AEDs on VDBP concentrations was not significant within the scope of this study.

Among the five identified significant associations, none were supported by the colocalization analysis, as all PP.H4.abf values were below 0.8 (Table S8). Additionally, LD Score Regression (LDSC) analysis did not reveal any genetic correlations (*p* < 0.05/10 after multiple testing correction) among the eQTLs of the five positive drug targets, and colocalization analysis confirmed a lack of genetic colocalization (Table S9). This suggests that these effects are likely independent within the genetic background.

The results of the sensitivity analyses are detailed in Table S10. Some genes had only one SNP available for analysis, which did not satisfy the criteria for robust sensitivity assessment. As in previous analyses, we utilized the IVW method with a random effects model to address the impact of heterogeneity on causal relationship estimates. Among the significant findings, the MR‐Egger intercept was close to zero, indicating no evidence of directional pleiotropy. The leave‐one‐out analysis revealed that no single SNP substantially distorted the results, suggesting that none of the SNPs significantly drove the observed effects. Furthermore, the global test performed by MR‐PRESSO found no evidence of horizontal pleiotropy.

## 4 Discussion

In this study, we investigated the relationships among serum 25(OH)D levels, VDBP, epilepsy, and the effects of AEDs. Our analyses, however, did not reveal any causal relationships between serum 25(OH)D levels, VDBP, and epilepsy. We identified five genes related to serum 25(OH)D levels, the majority of which were associated with lower levels. Inhibitors of SCN4A, CA13, and ALDH5A1, along with GABBR1 agonists, were significantly correlated with lower serum 25(OH)D levels, whereas CA8 antagonists were associated with an increase in serum 25(OH)D levels.

Numerous clinical studies have found that a substantial proportion of epilepsy patients exhibit insufficient VitD levels, with prevalence rates surpassing 40% in some reports [4, 5, 6, 7]. VitD levels have been proposed to affect the occurrence of epilepsy, potentially through hormonal or genetic mechanisms [36, 37]. Conversely, research indicates that during acute or exacerbated epilepsy episodes, disruptions in vitamin D3 metabolism lead to a rapid decline in plasma 25(OH)D3 levels [38]. This suggests that the onset of epilepsy may affect vitamin D levels. Thus, the causal relationship between VitD and epilepsy remains unclear.

Notably, previous MR studies have explored the impact of serum 25(OH)D on epilepsy, yielding inconsistent results. Some studies found no causal link [39], whereas others suggested that higher serum 25(OH)D levels might act as a protective factor for certain epilepsy subtypes [40, 41]. Additionally, previous research mainly concentrated on the unidirectional causal relationship between serum 25(OH)D and epilepsy. To address these gaps and offer a more comprehensive understanding, we utilized the most recent and largest epilepsy GWAS dataset, roughly twice the size of previous studies, to perform a bidirectional MR analysis. It is worth noting that in exploring the reverse causal relationship between vitamin D and epilepsy, certain subtypes were associated with very few significant SNPs. To address this, we relaxed the significance threshold, which may introduce some degree of weak instrument bias, and the inference of causality should be treated with caution.

Our MR results do not establish a causal link between serum 25(OH)D levels and epilepsy or its subtypes. Although a correlation was observed between increased genetically determined risk for focal epilepsy and elevated serum 25(OH)D levels, this association lacked statistical significance after correction. It is crucial to consider that VitD might display non‐linear relationships with certain diseases [42, 43, 44]. For instance, a recent MR study indicated that although linear analyses found no relationship between genetically predicted vitamin D levels and depression, stratification by vitamin D levels uncovered an association with lower levels [43]. This implies that VitD might also have a non‐linear relationship with epilepsy risk, where certain ranges of VitD levels could be associated with epilepsy risk. The GWAS data used in this study were not stratified, which limits the exploration of potential non‐linear associations. Future research utilizing individual‐level exposure and outcome data may be essential for thoroughly investigating these non‐linear associations.

To further explore the potential connections between epilepsy and serum 25(OH)D levels, we considered VDBP, which is closely related to VitD transport. A study by Leong et al. demonstrated that each standard deviation increase in VDBP is associated with a 5 nmol/L increase in 25(OH)D levels [10]. The GWAS data employed in this study had previously established a unidirectional causal relationship between genetically predicted VDBP levels and serum 25(OH)D levels [45] (OR = 1.117, 95%CI: 1.076–1.162, *p* = 1.41e−8), therefore, this relationship was not reanalyzed in our study. Our results indicate that the genetically predicted risk of JME is associated with lower VDBP levels, suggesting that patients with this subtype may exhibit reduced serum 25(OH)D levels as a consequence of lower VDBP levels.

Moreover, VDBP might also be related to seizure susceptibility [12]. In animal models of epilepsy, VDBP overexpression in the hippocampal region has been associated with heightened seizure sensitivity [46]. A candidate gene study suggested that VDBP polymorphisms may play a role in epilepsy susceptibility within the Han Chinese population [14]. To delve deeper into this issue, we employed GWAS data derived from blood samples to assess whether VDBP‐related SNPs, which have reached genome‐wide significance thresholds, are causally associated with epilepsy in European populations. However, no significant association was identified in our analysis. This discrepancy may be attributed to differences in tissue expression or population‐specific factors.

Notably, this study utilized GWAS data derived from a neonatal population, which requires consideration when interpreting the findings. Ethnicity, specific medications, pregnancy, and chronic diseases are recognized as factors influencing VDBP levels [47]; however, current evidence does not indicate a significant effect of age. Meanwhile, the GWAS dataset underwent genotype adjustments, effectively reducing the impact of VDBP polymorphisms. Furthermore, this dataset represents the largest sample size in VDBP research, far surpassing that of other studies [48, 49]. Additionally, its validation across neonatal and adult vitamin D levels, as well as autoimmune diseases, highlights its broader applicability [22]. Based on these considerations, we selected this GWAS dataset for MR analysis.

Recent research has identified that certain AEDs can contribute to decreased VitD levels in patients with epilepsy [15, 16, 17]. Specifically, liver enzyme‐inducing AEDs such as valproic acid and carbamazepine are associated with lower VitD levels [50, 51, 52], with the magnitude of this effect appearing to increase with the number of AEDs used [15, 16]. However, some studies have reported vitamin D deficiency or insufficiency in epilepsy patients even before starting AED treatment [17, 53]. Dong et al. [17] demonstrated that neither combination therapy with AEDs nor long‐term treatment with valproic acid was effective in maintaining reductions in baseline serum vitamin D levels in epilepsy patients. Therefore, there is ongoing debate about whether AEDs affect vitamin D levels.

Our study identified five AED target genes significantly associated with vitamin D levels, indicating an independent effect of Topiramate, Lamotrigine, Carbamazepine, Oxcarbazepine, Brivaracetam, Progabide, and Valproic Acid on reducing serum 25(OH)D levels. However, current research has not extensively explored the connections between these genes and VitD, with only limited supporting evidence available in the literature. For example, studies have reported that vitamin D deficiency is frequently observed in patients with familial hypokalemic periodic paralysis with SCN4A gene mutations, although the exact mechanism remains unclear [54]. Additionally, GABBR1 expression might affect vitamin D levels indirectly by modulating parathyroid hormone secretion through CaSR‐mediated calcium sensing in the parathyroid gland [55]. Due to the lack of direct evidence, further research is needed to clarify the mechanisms underlying these associations and to better understand how AEDs impact vitamin D levels.

Furthermore, after applying multiple corrections, no significant association was observed between the use of AEDs and VDBP levels. This implies that these medications may not significantly alter VDBP concentrations in the blood. These findings align with previous research by Välimäki et al. [56], which reported that serum vitamin D binding protein levels remain normal in epilepsy patients treated with carbamazepine or phenytoin.

## 5 Limitations

Despite the comprehensive analysis conducted in our study, several limitations should be acknowledged. First, while MR helps mitigate the impact of confounding factors, residual confounding may still affect our results. Second, the data used in this study, such as serum VitD levels, were not stratified, and thus, only overall phenotypic GWAS data were analyzed, potentially missing non‐linear associations. Third, although we consider the VDBP GWAS dataset to have reasonable applicability, the reliability of extrapolating genetic data from newborns to adult populations remains uncertain. Future studies should incorporate adult GWAS datasets and comparative analyses for validation. Fourth, some epilepsy subtypes had relatively small sample sizes, potentially limiting genetic power and the robustness of the findings. Larger, well‐powered studies are necessary to validate the associations observed and to provide subtype‐specific insights. Furthermore, not all drug targets for AEDs are present in the eQTLGen database, which may result in incomplete representation of the genetic variants associated with certain drugs. Additionally, MR studies typically examine the effects of lifetime exposure, whereas drug exposure typically occurs over shorter periods. Consequently, the effect sizes estimated in our study may not fully capture the association between critical exposure periods and outcomes. Moreover, the drug target gene models employed in our analysis address only specific treatment targets, potentially neglecting the pharmacokinetics of drug use. These limitations underscore the necessity for further research to address these constraints and deepen our understanding of the relationship between epilepsy risk and VitD and VDBP.

## 6 Conclusions

This study did not establish a causal relationship between genetically determined serum 25(OH)D levels, VDBP and epilepsy or its subtypes. Among AEDs, significant associations were identified between five drug target genes and serum 25(OH)D levels, with the majority showing reductions. Specifically, medications including Topiramate, Lamotrigine, Carbamazepine, Oxcarbazepine, Brivaracetam, Progabide, and Valproic Acid were linked to reduced serum 25(OH)D levels. No evidence supported a connection between AEDs and VDBP levels. These findings contribute to our understanding of the interplay between epilepsy, vitamin D, and vitamin D binding protein, highlighting the need for further research to elucidate these complex interactions and their implications for clinical management.

## Author Contributions

Z.C. designed the study, conducted the analysis, and wrote the manuscript. J.Z. contributed to the study design, data interpretation, and manuscript revision. X.P. assisted with data collection and analysis. H.Z. supported statistical analysis and results interpretation. W.S. contributed to study design and data interpretation. G.L. provided input on data interpretation and manuscript revision. Y.G., the corresponding author, supervised the study, revised the manuscript. All authors have read and agreed to the published version of the manuscript.

## Conflicts of Interest

The authors declare no conflicts of interest.

## Supporting information


Table S1.

Table S2.

Table S3.

Table S4.

Table S5.

Table S6.

Table S7.

Table S8.

Table S9.

Table S10.

## Data Availability

The data that supports the findings of this study are available in the supporting material of this article.
